# Death by Disimpaction: A Bradycardic Arrest Secondary to Rectal Manipulation

**DOI:** 10.1155/2016/5489325

**Published:** 2016-12-28

**Authors:** Christopher S. Sampson, Cory M. Shea

**Affiliations:** Department of Emergency Medicine, University of Missouri-Columbia, One Hospital Drive, DC 029.1, Columbia, MO 65212, USA

## Abstract

Rectal examination and fecal disimpaction are common procedures performed in the Emergency Department on a daily basis. Here, we report a rare case of a patient suffering a cardiac arrest and ultimately death likely due to rectal manipulation. A 66-year-old male presented to the Emergency Department (ED) with a complaint of abdominal distention and constipation. A rectal exam was performed. During the examination the patient became apneic. On the cardiac monitor the patient was found to be in pulseless electrical activity with a bradycardic rate. Our recommendation would be to provide adequate analgesia and close patient monitoring of those undergoing this procedure especially patients with significant stool burdens.

## 1. Introduction

Rectal examination and fecal disimpaction are procedures performed in the Emergency Department (ED) on a daily basis. Digital exploration is a necessary part of a patient's evaluation in multiple patient presentations, including gastrointestinal (GI) bleeding, evaluation of the prostate, or performing disimpaction. The procedure, while uncomfortable, is generally considered benign and is rarely associated with serious complications. Complications are rare but may include perforation, anal fissure formation, urinary tract obstruction, and rectal bleeding.

Fatal arrhythmias have been reported in the elderly. Because disimpaction often occurs in the elderly and the chronically ill, complications can have fatal results [1, 2]. Bradycardic pulseless electrical activity (PEA) arrest is a clinical condition frequently encountered in the ED and one that often represents a terminal event. Here, we report a rare case of a patient suffering a cardiac arrest and ultimately death likely due to rectal manipulation. The arrest appeared to have been precipitated by a vagal-mediated bradycardic event in the setting of concurrent rectal manipulation and after a research of the literature this would be the first reported case of its kind.

## 2. Case Report

A 66-year-old male who was a long standing inmate in a forensic psychiatric hospital presented to the Emergency Department with a complaint of abdominal distention. The patient denied any nausea or vomiting but reported a decreased appetite which he attributed to abdominal discomfort. His last bowel movement was five days ago. The patient also endorsed dysuria. A computerized tomography (CT) scan of the abdomen and pelvis was performed prior to ED arrival and the images were brought with patient.

The patient had a past medical history notable for paranoid schizophrenia, bipolar disorder, latent tuberculosis, and ascending aortic aneurysm. There was no known history of liver disease. The patient's medications included clozapine, fluphenazine, lithium, paroxetine, lorazepam, and haloperidol. He had no known drug allergies and had no recent medication changes. Complete review of systems was otherwise negative. The patient had no history of previous surgeries, did not smoke, drink, or consume illicit drugs, and had a noncontributory family history.

On initial presentation to the ED his vital signs were heart rate (HR), 104; blood pressure (BP), 147/101; respiratory rate (RR), 22; pulse oximetry, 93% on room air; and temperature, 36.4 degrees Celsius.

On examination of the patient, his oral mucosa was noticeably dry. His cardiac exam was unremarkable with normal S1 and S2, no murmurs, rubs, or gallops, and 2+ pulses in all four extremities and his lungs were clear to auscultation bilaterally. The patient's abdomen was firm and grossly distended and was noted to resemble a full-term pregnant patient. Dilated superficial veins were noted on the abdomen and palpation revealed mild, diffuse tenderness without rebound or guarding. The patient was awake, alert, and orientated to time, date, and environment.

**Figure 1:**
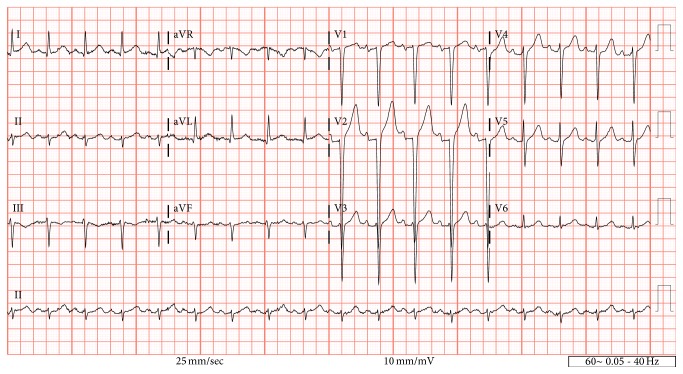
Electrocardiogram.

Labwork was notable for sodium of 115 mmol/L and a serum osmolality of 251 mOsm/kg. The potassium level was 5.2 mmol/L, chloride 80 mmol/L, bicarbonate 21 mmol/L, creatinine 1.58 mg/dL, lactic acid 1.5 mmol/L, troponin T < 0.01 ng/mL, and lithium level 1.0 mmol/L. The patient was mildly anemic with a hemoglobin of 11.5 g/dL, while his remaining complete blood count (CBC) was unremarkable with a white blood cell (WBC) count of 9.44/L and platelets of 318/L. An electrocardiogram was obtained during the ED evaluation (Figure 1) which showed a sinus tachycardia of 105 beats per minute, QRS 98s, QTc 415 ms, and peaked T waves in the precordial leads.

**Figure 2:**
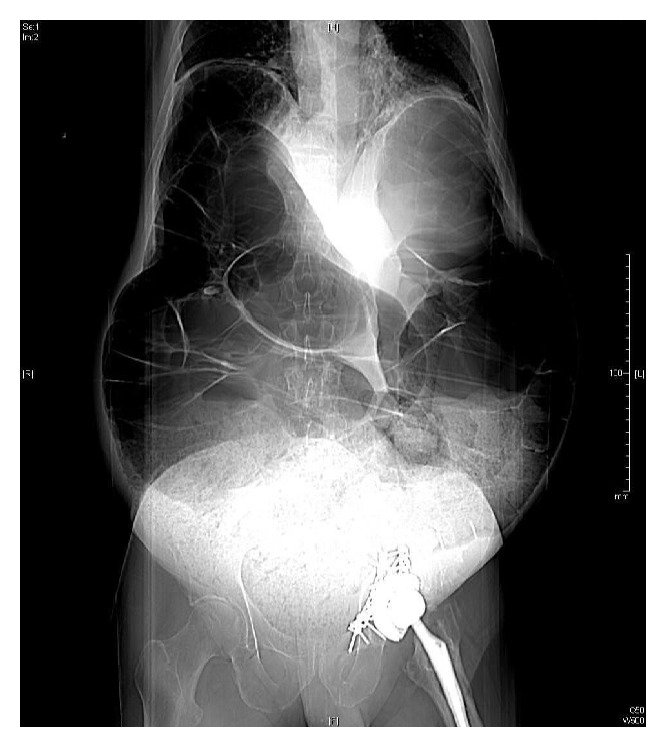
CT scout film showing dilated loops of bowel.

**Figure 3:**
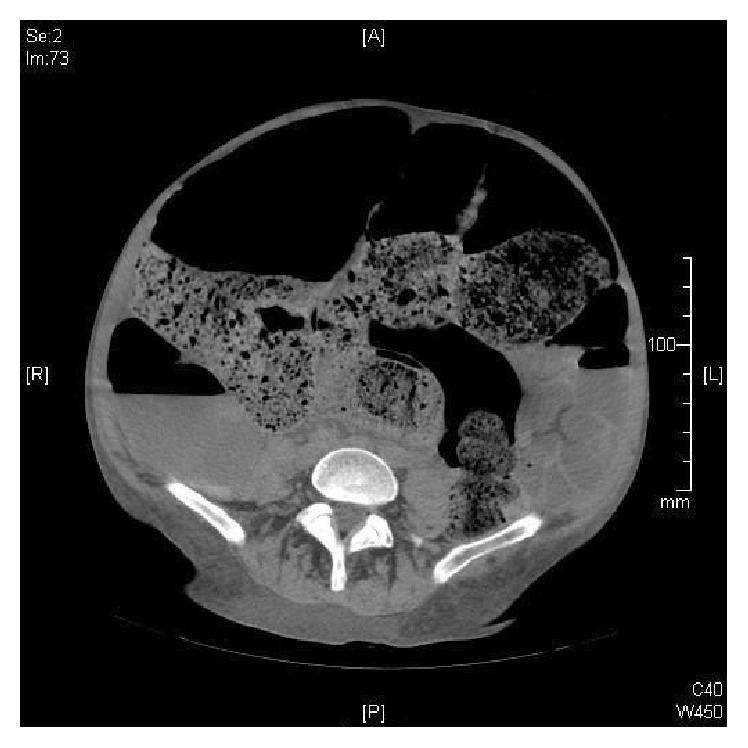
CT cut showing dilated colon and large stool burden.

Upon review of the CT, large dilated loops of colon were noted up to 12 cm (Figures 2 and 3).

During the ED course, the acute care surgical service was consulted for further management. A rectal exam was performed by the resident physician with a plan for disimpaction immediately following. The patient was positioned on his left side. Soft stool was found on rectal exam. During the rectal examination, the nurse in the room noted that the patient was apneic and a code was called. The patient was unresponsive for less than one minute prior to the nurse noticing his status change. The patient had no new complaints prior to his arrest.

Unfortunately, the patient was not on a cardiac monitor at this time. Upon immediately placing the patient on a monitor, a bradycardic rate was noted. Following a pulse check, the patient was then found to be in pulseless electrical activity. Cardiopulmonary resuscitation (CPR) was started and advanced cardiac life support (ACLS) protocol was followed including intubation. Initial bedside ultrasound showed no cardiac activity. Following multiple rounds of CPR and ACLS medications, return of spontaneous circulation (ROSC) was obtained. The patient then again went into PEA with a bradycardic rhythm but ROSC was again obtained and remained. A postarrest arterial blood gas was notable for pH 6.84, pCO_2_ 100 mmHg, and pO_2_ 94.5 mmHg. The patient was then admitted to Intensive Care Unit (ICU) on epinephrine, norepinephrine, and vasopressin infusions. At time of ICU admission the patient was in a normal sinus rhythm of 98. Eight days later the patient expired following confirmation of anoxic brain injury and a decision made to provide comfort care.

## 3. Discussion

A search of the literature through PubMed could find no reported cases of death secondary to rectal examination or disimpaction. Fausel and Paski reported a case of autonomic dysreflexia in a patient that led to a seizure following a colonoscopy [3]. Ruan reported a case of a 29-year-old male who converted from atrial fibrillation to normal sinus rhythm following rectal examination prior to anticoagulation initiation [4]. We believe that, given the situation, this patient likely suffered a cardiac arrest secondary to bradycardic stimulation from rectal manipulation causing an increase in parasympathetic activity as mediated by the vagus nerve. Any stimulation of this nerve along its pathway leads to a release of acetylcholine from the nerve endings in the heart causing a parasympathetic response [5]. Given this patient's significant hyponatremia and hyperkalemia, the increased vagal stimulation may have been the tipping point in an already tenuous situation. The patient's medications also may have played a role in the patient's cardiac arrest. Lithium is known to have cardiac affects including T wave flattening, sinus node dysfunction, ventricular fibrillation, and ventricular tachycardia [6, 7]. These complications are often worse in toxic ranges but there is a case report by Oudit et al. of a patient suffering sinus node arrest in the setting of a therapeutic lithium level. It is thought that this patient had subclinical sinus node dysfunction that was worsened by lithium-induced cardiac sodium channel blockade [8]. This case illustrates that what we believe is a rare but potential risk in patient undergoing disimpaction. Our recommendation would be to provide adequate analgesia and close patient monitoring of those patients undergoing this procedure who may require more than usual rectal manipulation due to significant stool burdens. Special consideration should also be applied to patients who are receiving increased rectal manipulation that have known electrolyte abnormalities and are on medications that may predispose them to fatal arrhythmias.
